# A Pseudopterane Diterpene Isolated From the Octocoral *Pseudopterogorgia acerosa* Inhibits the Inflammatory Response Mediated by TLR-Ligands and TNF-Alpha in Macrophages

**DOI:** 10.1371/journal.pone.0084107

**Published:** 2013-12-16

**Authors:** Yisett González, Deborah Doens, Ricardo Santamaría, Marla Ramos, Carlos M. Restrepo, Luciana Barros de Arruda, Ricardo Lleonart, Marcelino Gutiérrez, Patricia L. Fernández

**Affiliations:** 1 Centro de Biología Celular y Molecular de Enfermedades, Instituto de Investigaciones Científicas y Servicios de Alta Tecnología, Ciudad de Panamá, Panamá; 2 Department of Biotechnology, Acharya Nagarjuna University, Guntur, India; 3 Centro de Descubrimiento de Drogas y Biodiversidad, Instituto de Investigaciones Científicas y Servicios de Alta Tecnología, Ciudad de Panamá, Panamá; 4 Laboratorio de Genética e Imunologia das Infecções Virais, Departamento de Virologia, Instituto de Microbiologia, Universidade Federal do Rio de Janeiro, Rio de Janeiro, Brasil; University of Bergen, Norway

## Abstract

Several diterpenoids isolated from terrestrial and marine environments have been identified as important anti-inflammatory agents. Although considerable progress has been made in the area of anti-inflammatory treatment, the search for more effective and safer compounds is a very active field of research. In this study we investigated the anti-inflammatory effects of a known pseudopterane diterpene (referred here as compound **1**) isolated from the octocoral *Pseudopterogorgia acerosa* on the tumor necrosis factor- alpha (TNF-α) and TLRs- induced response in macrophages. Compound **1** inhibited the expression and secretion of the inflammatory mediators TNF-α, interleukin (IL)-6, IL-1β, nitric oxide (NO), interferon gamma-induced protein 10 (IP-10), ciclooxygenase (COX)-2, inducible nitric oxide synthase (iNOS) and monocyte chemoattractant protein-1 (MCP-1) induced by LPS in primary murine macrophages. This effect was associated with the inhibition of IκBα degradation and subsequent activation of NFκB. Compound **1** also inhibited the expression of the co-stimulatory molecules CD80 and CD86, which is a hallmark of macrophage activation and consequent initiation of an adaptive immune response. The anti-inflammatory effect was not exclusive to LPS because compound **1** also inhibited the response of macrophages to TNF-α and TLR2 and TLR3 ligands. Taken together, these results indicate that compound **1** is an anti-inflammatory molecule, which modulates a variety of processes occurring in macrophage activation.

## Introduction

Inflammation is a host response triggered by exogenous stimuli, such as infections, or by stimuli from endogenous sterile injuries. It is characterized by the recruitment and accumulation of immune cells in injured sites and by the production of soluble mediators including reactive oxygen and nitrogen species, chemokines, lipid mediators and cytokines. These mediators are essential for controlling the inflammation and tissue repair, but may also exacerbate tissue damage. The inflammatory response is initiated by cellular sensing of either Pathogen-Associates Molecular Patterns (PAMPs) or Damage-Associated Molecular Patterns (DAMPs) through Pattern Recognition Receptors (PRR), such as Toll-Like Receptors (TLRs) and NOD-Like Receptors (NLRs), which trigger specific signaling pathways [1].

Macrophages are one of the most important cells implicated both in the resolution and exacerbation of inflammation, depending on the stimuli and the pattern of the elicited immune response. Activation of macrophages by PRRs or by cytokine receptors, such as TNF-α and IL-1β receptors (TNFR and IL1R respectively) leads to the production of inflammatory mediators such as NO, TNF-α, IL-1β, IL-6 and COX-2. Macrophage-derived NO is produced by iNOS and may be beneficial due to its immunomodulatory, anti-tumoral and anti-pathogenic effects. However, high and sustained levels of NO are detrimental to the host and are involved in the pathogenesis of several diseases [2]. Inflammatory conditions are also associated with high levels of TNF-α and IL-1β, which have an autocrine and paracrine effect on immune cells, potentiating the inflammatory response.

Intracellular signaling pathways triggered by PRRs in different cell types, including macrophages, culminate in the activation and nuclear translocation of NFκB, which induces the expression of most of the mediators mentioned above [3,4]. NFκB activation also regulates the transcription of several genes implicated in apoptosis, proliferation, cellular adhesion, stress response and tissue remodeling [5]. Due to its central role in the inflammatory response NFκB is involved in many human pathological conditions, including acute and chronic inflammation, and thus constitutes a suitable target for the development of new anti-inflammatory drugs. 

The NFκB family consists of five proteins (RelA/p65, cRel, RelB, p50, p52) that form homo- and heterodimers depending on what genes need to be regulated. Among these, p65:p50 is the major complex formed after cellular activation by microbial products and pro-inflammatory cytokines [6]. NFκB activation is controlled by the IKK complex, which induces the phosphorylation and degradation of IκB inhibitor proteins, allowing the nuclear translocation of these transcription factors [7]. 

Most drugs used today for the treatment of disease are derived from natural products [8]. Studies in terrestrial organisms have been extended to marine organisms, which have enormous potential as a source of novel active compounds [9-12]. Among marine organisms, gorgonian octocorals are a well-known source of natural bioactive products. A group of compounds frequently found in octocorals are the diterpenes, which possess a wide range of biological activities including antibacterial, antiviral, antifungal, antitumor, anti-inflammatory and antiprotozoal properties [13,14]. Some coral diterpenes have been identified as modulators of NFκB signaling pathways [15-17]. 

An interesting group of diterpenes restricted to the marine environment are the pseudopteranes: a family composed of approximately 30 members. Despite their ubiquity, the biological properties of the majority of these diterpenes have not been extensively explored [18]. Two of these compounds, the pseudopterolide and the kallolide A, have been reported to reduce the inflammatory response in a PMA-induced topical inflammation assay [19]; however, the mechanisms involved in this effect were not described. 

**Figure 1:**
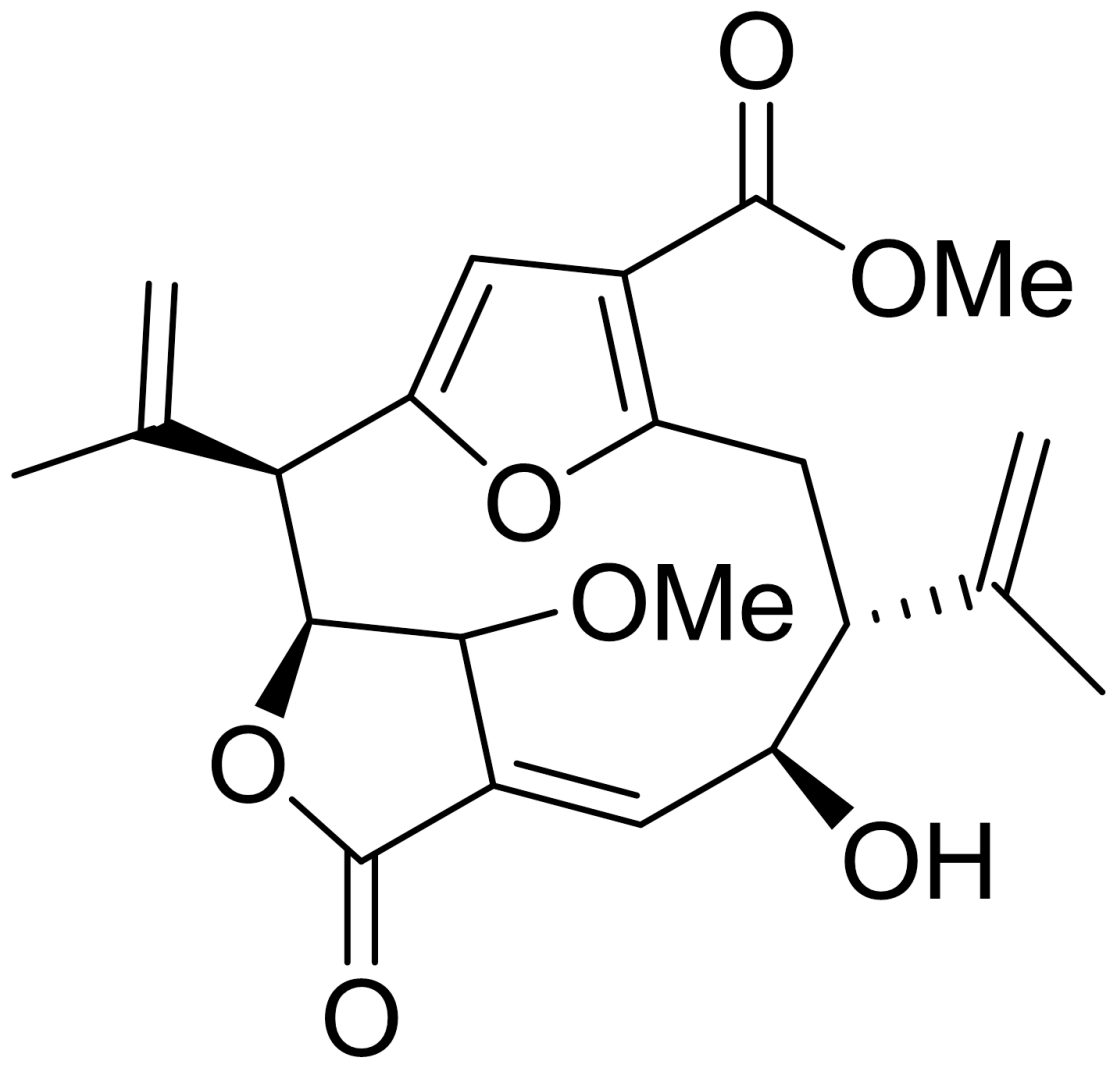
Schematic representation of compound 1.

We investigated the anti-inflammatory activity of a pseudopterane diterpene (compound **1**) (Figure 1), isolated from the octocoral *Pseudopterogorgia acerosa* collected in Bocas del Toro, Panama. Compound **1** was previously described as a methanol adduct of the diterpene pseudopterolide [20] as a consequence of the storage of the coral specimens in methanol. Another research group later isolated compound **1** in the absence of methanol [21] suggesting that this metabolite is a natural product produced by *P.acerosa*. We observed that compound **1** has a potent anti-inflammatory activity. Compound **1** inhibited the expression and secretion of several inflammatory mediators including TNF-α, IL-6, IL-1β, NO, IP-10, COX2 and MCP-1 induced by LPS in primary murine macrophages cultures. The anti-inflammatory effect observed was due to the inhibition of IκBα degradation and subsequent suppression of p50 and p65 activation. Compound **1** also inhibited the secretion of proinflammatory cytokines induced by TNF-α and by TLR2 and TLR3 ligands, and reduced the expression of co-stimulatory molecules (CD80 and CD86) induced by LPS. We also compared the anti-inflammatory activity of compound **1** with that of isogorgiacerodiol (Figure S1) isolated from the same coral preparation and previously described by Tinto et al. in 1991 [21]. We demonstrated that compound **1** has a higher anti-inflammatory activity than isogorgiacerodiol. These results indicate that compound **1** is a potential molecule for the development of new anti-inflammatory drugs.

## Materials and Methods

### Mice

Female and male C57BL/6 mice, 8 weeks of age, were obtained from INDICASAT’s mice facility. Mice were purchased from Harlan Laboratory S.A. de C.V. and stored at INDICASAT. The animals were kept at a constant temperature (25 °C) with free access to food and water in a room with a 12 hours (h) light/dark cycle. 

### Ethics Statement

All experiments were performed in strict accordance with guidelines from the Institutional Animal Welfare Committee and the Guide for the Care and Use of Laboratory Animals of the National Institutes of Health. The protocol was also approved by the Institutional Animal Care and Use Committee of INDICASAT-AIP. 

### Reagents

LPS 0111:B4 from *E. coli*, synthetic bacterial lipopeptide (Pam_3_CysSerLys_4_), Poly I:C, were obtained from InvivoGen (San Diego, CA). Recombinant mouse TNF-α was from R&D Systems, Inc. (Minneapolis, MN). RPMI medium and fetal bovine serums (FBS) for macrophage culture were obtained from Gibco (Grand Island, NY). DMSO was obtained from Sigma-Aldrich (St. Louis, MO).

### Biological material collection and identification

The octocoral *Pseudopterogorgia acerosa* (Order Gorgonacea, Family Gorgonidae) was collected by hand using SCUBA at 4.5 m depth in Bastimentos National Park, located in the Caribbean off the coast of Bocas del Toro, Panama in November 2009. Permission to collect the coral used in this study was issued by Autoridad Nacional del Ambiente (ANAM, Government of Panama, permit #: SC/A-30-09). The coral specimen was identified as *Pseudopterogorgia acerosa* (Pallas) based on its morphology and SEM-micrographs of the coral sclerites in the Smithsonian Tropical Research Institute. A reference specimen was deposited at INDICASAT’s Center for Biodiversity and Drug Discovery (CBDD) under the number GLBO-241109-03. 

### Isolation and characterization of compounds

The organism (958.8 g) was minced and exhaustively extracted with CH_2_Cl_2_ and MeOH. The organic extract was evaporated *in vacuo* to give 20.5 g of a dark oily residue. The CHCl_2_-MeOH extract (20.0 g) was chromatographed by column chromatography on silica gel and eluted with a stepwise gradient of 0 %–100 % EtOAc in hexanes followed by 0 %–100 % MeOH in EtOAc to yield 10 fractions (A–J). Fraction H (1.0 g) was purified by a second column chromatography eluted with a gradient of 6.3 %-70 % EtOAc in CH_2_Cl_2_ followed by 100 % acetone and 100 % methanol to yield 17 fractions (H1-H17). Fraction H-7 yielded 35.2 mg of a pure pseudopterane diterpene (1) [20]. Fraction F was concentrated (349 mg) and further chromatographed on silica gel eluted with a stepwise gradient of 50 %, 70 %, 100 % CH_2_Cl_2_ in hexanes, followed by 5 %, 10 %, 20 %, 30 %, 50 %, 70 %, 100 % of EtOAc in CH_2_Cl_2_ and 10 % MeOH in EtOAc to yield 21 fractions (F1-F21). Fraction F19 was further purified by HPLC (5 µm Silica gel Sphereclone column eluted with a gradient of 40 %–100 % EtOAc in hexanes in 80 min at 1.0 mL/min) to yield 12 fractions denoted I-XII. Fraction XI (13.3 mg) was re-injected in HPLC (5 µm Silica gel Sphereclone column eluted with a gradient of 75 %–100 % EtOAc in hexanes in 75 min at 1.0 mL/min) to yield 6 fractions (XIA-XI-F). Fraction XIE contained 1.1 mg of the diterpene isogorgiacerodiol [21]. Structural determination of both compounds was carried out by comparing their spectroscopic data (^1^H-NMR, ^13^C-NMR and HRMS) and optical rotations with those reported in the literature [20,21].

### Macrophage culture

Peritoneal macrophages were obtained five days after i.p. instillation of 2 mL of thioglycollate 3 %, by peritoneal washing with chilled RPMI. Cells were seeded in RPMI with 10 % FCS at 2x10^5^/well in 96-well plates for cytokine determination, 2.5x10^6^/well or 4x10^6^/well in 6-well plates for western blot and mRNA analysis respectively and cultured for 2 h at 37 °C in an atmosphere of 5 % CO_2_. In all cases non-adherent cells were removed by washing and adherent cells were stimulated as indicated in figure legends. Briefly, for dose- response experiments cells were treated with different concentrations of compound **1** (2.5, 5, 12.5, 25 or 50 μM) 1 h previous to the stimuli with 10 ng/mL of LPS, 100 ng/mL of Pam_3_Cys or 20µg/mL of Poly I:C. Supernatants were collected 24 h after the stimulus and NO, TNF-α, IL-6, IL-1β or IP-10 concentrations were determined by ELISA. For western blot and mRNA analyses cells were treated with 25 μM of compound **1** and stimulated with LPS (100 ng/mL and 1 μg/mL, respectively). Cell extracts were harvested at the time points indicated in figure legends and run in SDS-PAGE for western blot or used for RNA isolation for mRNA analysis. 

Bone marrow derived macrophages (BMDM) were obtained after differentiation of cells from murine femur and tibia. The cells were cultured in a concentration of 4x10^6^/10 mL in RPMI containing 20 % FCS and 30 % L929 cell culture supernatant at 37 °C in an atmosphere of 5 % CO_2_. After 4 days fresh medium was added and macrophages were collected at day 7. Adherent cells (1x10^6^/well) were plated in 12-well plates in RPMI medium supplemented with 10 % FCS and maintained at 37 °C an atmosphere of 5 % CO_2_. Cells were stimulated with LPS (1 μg/mL) in the presence or absence of compound **1** (25 μM) for 24 h and the expression of CD80 and CD86 was evaluated by flow cytometry. 

All negative controls and stimulus were performed in the presence of 0.5 % DMSO since compound **1** is solubilized in DMSO. The known inhibitor of iNOS, the L-N6-(1-lminoethyl) lysine dihydrochloride (L-N6), was used as an inhibition control to test the cell-based assay.

### Cytokine measurements

Peritoneal macrophages were cultured as previously described. The concentrations of TNF-α, IL-6, IL-1β and IP-10 were determined by ELISA (DuoSet kit, R&D System, Inc. Minneapolis, MN), according to the manufacturer’s protocol. 

### NO measurements

The accumulation of nitrite in cell supernatants was measured as an indicator of NO production based on a Griess assay. The concentration of nitrite was determined by the Griess Reagent System (Promega, Madison, WI) according to the manufacturer’s protocol. 

### Flow cytometry

The cells were harvested after 24 h of the stimulus, washed with phosphate-buffered saline (PBS), and blocked with 200 µL 1 % BSA in PBS for 15 min. The cells were washed and then incubated with (5 μg/mL) of anti-mouse CD11b FITC, anti-mouse CD86 PE-Cy5 and/or anti-mouse CD80 APC (eBioscience, SanDiego, CA) diluted in 1 % BSA in PBS, for 30 min at 4 °C. After several washes, the cells were resuspended in PBS and analyzed by flow cytometry. Event acquisition was performed with a Partec CyFlow^®^ cytometer and the data were analyzed using FlowMax software (PARTEC, Münster, Germany) and FCS Express 4 Flow Cytometry (De Novo software, Los Angeles, CA). 

### Quantitative Real Time RT-PCR

Total RNA from elicited peritoneal macrophages was extracted using TRIzol (Life Technology Corporation: Invitrogen and Applied Biosystems, Carlsbad, CA), and 2 µg were reverse-transcribed using a High-Capacity cDNA reverse transcription kit (Life Technology Corporation: Invitrogen and Applied Biosystems, Carlsbad, CA). Subsequent quantitative real time PCR analysis was performed on an ABI 7500 (Applied Biosystems) using SYBR Green master mix (Applied Biosystems). Amplification conditions were as follows: 95 °C (10 min), 40 cycles of 95 °C (15 s), and 60 °C (60 s). All data were normalized to the corresponding HPRT expression, and the difference relative to the control level was shown. Analyses of relative gene expression data were performed by the 2^-∆∆^CT method. The primers sequences are TNF forward 5’-GGTCCCCAAAGGGATGAGAAGTTC- 3’ and reverse 5’-CCACTTGGTGGTTTACTACGACG- 3’; IL-6 forward 5’-TCATATCTTCAACCAAGAGGTA-3’ and reverse 5’-CAGTGAGGAATGTCCACAAACTG-3’; IL-1β forward 5’- GTAATGAAAGACGGCACA CC-3’ and reverse 5’-ATTAGAAACAGTCCAGCCCA-3’; HPRT forward 5’-GCTGGTGAAAAGGACCTCT- 3’ and reverse 5’-CACAGGACTAGAACACCTGC- 3’; IP-10 forward 5’-GAAATCCATCCCTGCGAGCCT-3’ and reverse 5’-TTGATGGTCTTAGATTCCGGATTC -3’; iNOS forward 5’-CCTCCACCCTACCAAGT-3’ and reverse 5’-CAGCTCCAAGGAAGAGTGA-3’; COX-2 forward 5’-CGTGGTCACTTTACTACGAG-3’ and reverse 5’-AGGTACATAGTAGTCCTGAGC-3’; MCP-1 forward 5’-CAGCAGGTGTCCCAAAGAAG-3’ and reverse 5’-GACCTTAGGGCAGATGCAGT-3’.

### Western blot analysis

Elicited peritoneal macrophages (2.5x10^6^ cells/well) were plated in 6-well plates. Non-adherent cells were removed after 2 h and adherent cells were stimulated as indicated in the figure legends. Cells were lysed in a buffer consisting of Tris-HCl (50 mM), NaCl (150 mM), NP40 (1 %), sodium deoxycholate (0.25 %), EDTA (1 mM), aprotinin (5 μg/mL), leupeptin (5 μg/mL), pepstatin (5 μg/mL), PMSF (1 mM), sodium orthovanadate (1 mM) and NaF (1 mM), pH 7.5. Twenty micrograms of protein diluted in loading buffer were boiled and subjected to electrophoresis in SDS-polyacrilamide gel (12 %) under reducing conditions. The proteins were transferred to a PVDF membrane at 4 °C for 2 hours. Membranes were then blocked with Tris-buffered saline solution with 0.05 % of Tween-20 (TBS-T) and 5 % fat free milk or TBS-T with 3 % of BSA for p-JNK detection. The membranes were incubated overnight with anti-phospho (p)-ERK1/2 (1/1000), anti-p-p38 (1/1000) (Cell Signaling Technology, Inc. Boston, MA) or 48 h with anti-p-JNK (1/1000) (Santa Cruz Biotechnology, Inc. Santa Cruz, CA) diluted in the blocking solution. The membranes were washed in TBS-T and incubated for 2 h with horseradish peroxidase-conjugated goat anti-rabbit (1/10000) or goat anti-mouse (1/10000) IgG polyclonal antibodies (Santa Cruz Biotechnology, Inc. Santa Cruz, CA). Specific bands were detected by chemiluminescence, using ECL substrate (Santa Cruz Biotechnology, Inc. Santa Cruz, CA). The normalization was performed by stripping membranes during 30 min at 50 °C in stripping buffer (β-mercaptoethanol 100 mM, SDS 2 %, Tris-HCl 62.5 mM, pH 6.7). After stripping, membranes were washed with TBS-T, blocked with 5 % fat free milk TBS-T, incubated overnight with rabbit anti-ERK2 (1/1000) (Santa Cruz Biotechnology, Inc. Santa Cruz, CA) and detection was performed as described above. The degradation and phosphorylation of IκBα protein was analyzed by western blot with polyclonal rabbit anti-IκBα (Sigma Aldrich, St. Louis MO) or monoclonal mouse phospho-IκBα (Ser32/36) (Cell Signaling Technology, Inc. Boston, MA) diluted (1/1000) in blocking solution. Detection of β-actin was used as loading control. 

### NFκB activation

The NFκB activation was determined using the Transcription Factor Assay Kit, Trans AM^TM^ NFκB family (Active Motif, Carlsbad, CA). Cells (3x10^6^/well) were stimulated with LPS in the presence or absence of compound **1**, at different time intervals as described in the figure legends. The preparation of nuclear extracts and measurement of NFκB activation was performed as recommended by the manufacturer. 

### MTT assay

After the removal of supernatants, 100 µl of MTT (Sigma Aldrich) (0.5 mg/mL) dissolved in RPMI was added to each well and cells were incubated ON at 37 °C. The supernatants were removed and formazan crystals were dissolved in 100 µl of 0.04 M HCl in isopropanol. The color was analyzed at 570 nm using an ELISA plate reader. The percent of viable cells was calculated using the formula: % viability: [(OD sample) x 100 %]/ (OD control). The non-stimulated cells and cultured in medium plus 10 % FCS and 0.5 % DMSO represented 100 % viability.

### Statistical Analysis

Data are presented as means ± S.E.M. Results were analyzed using a statistical software package (GraphPad Prism 5). Statistical analyses were performed by Student’s t test or one-way ANOVAs followed by post hoc Tukey test. A difference between groups was considered to be significant if *P* < 0.05 (*, *P* < 0.05; **, *P* < 0.01). Inhibitory concentration 50 % (IC_50_) values were calculated adjusting a sigmoidal dose-response curve following GraphPad Prism 5 procedure.

## Results

### Compound 1 inhibits the production of TNF-α and IL-6 induced by LPS in murine peritoneal macrophages

**Figure 2:**
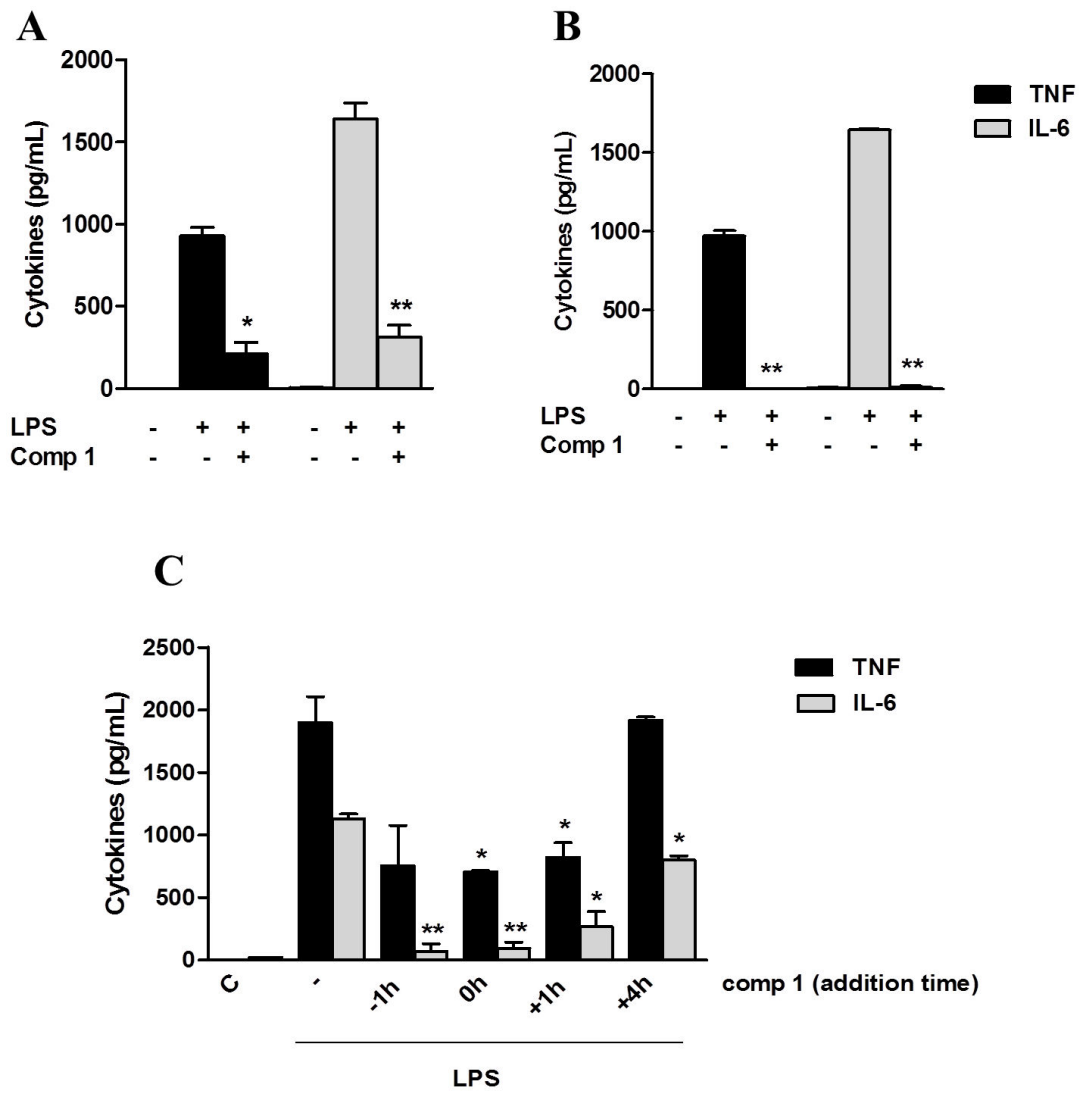
Compound 1 does not appear to interfere with LPS-TLR4 interaction. (A, B) Peritoneal macrophages were treated with 25 μM of compound **1**. After 1 hour, compound **1** was either removed (A) or not (B) from the supernatant and cells were stimulated with LPS (10 ng/mL). (C) Macrophages were treated with compound **1** (25 μM) 1 h before, at the same time, 1 h after or 4 h after LPS (10 ng/mL) stimulation. Supernatants were collected 6 h after the stimulus with LPS and TNF-α (black bars) and IL-6 (gray bars) concentrations were determined by ELISA. Results represent means ± S.E.M. from stimuli performed in duplicates and are representative of two different experiments. *, *P* ˂ 0.05; **, *P* ˂ 0.01, compared with LPS stimulus alone.

LPS is a component of the outer membrane of Gram-negative bacteria and is the most studied TLR4 ligand. Signaling induced by LPS through TLR4 leads to the activation of NFκB and the production of inflammatory mediators. In order to evaluate the anti-inflammatory activity of compound **1**, we analyzed its ability to modulate the secretion of TNF-α and IL-6 pro-inflammatory cytokines by macrophages cultured with LPS. Primary cultures of macrophages were treated with compound **1**, which was either removed from the culture after one hour or not, and then stimulated with LPS (Figure 2, A and B). We observed that compound **1** suppressed the cytokine production induced by LPS in macrophages even when it was removed from the culture medium before the LPS treatment (Figure 2A), indicating that it is not acting by sequestering the LPS out of the supernatant. 

We also performed a kinetic experiment by adding compound **1** at different periods before and after LPS addition. A significant inhibition of TNF-α and IL-6 secretion was observed when the compound was added 1 h and 4 h after LPS-treatment, respectively, suggesting that its inhibitory effect may involve mechanisms associated to signaling steps downstream from LPS recognition by TLR4 (Figure 2C). 

Compound 1 inhibits the production of several pro-inflammatory mediators induced by LPS at protein and mRNA levels

**Figure 3:**
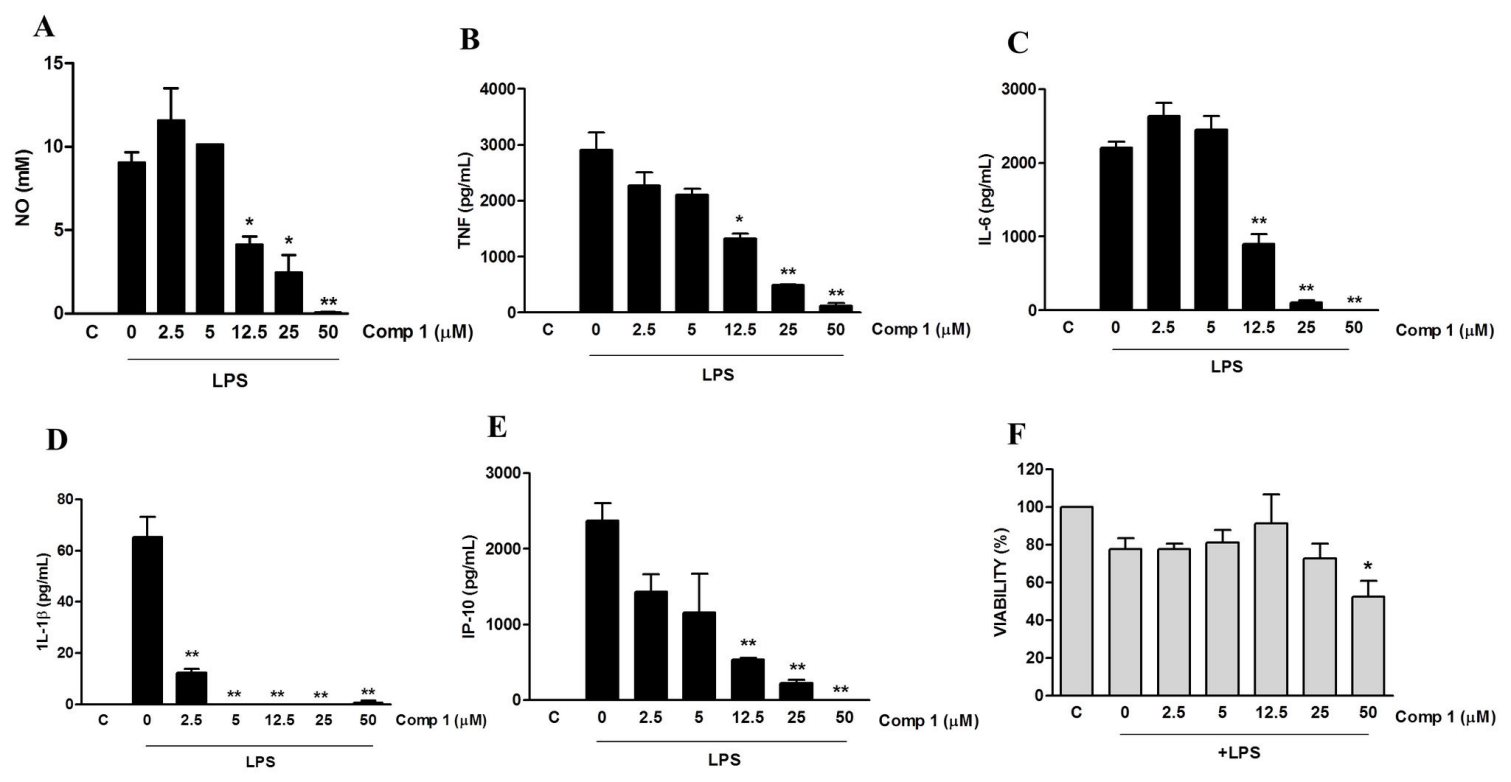
Compound 1 inhibits the production of pro-inflammatory mediators induced by LPS in macrophages. Peritoneal macrophages were treated with the indicated concentrations of compound **1** (2.5, 5, 12.5, 25 or 50 μM). After 1 hour cells were stimulated with 1 μg/mL (A) or 10 ng/mL (B-F) of LPS. Supernatants were collected 24 h after the stimulus and NO (A), TNF-α (B), IL-6 (C), IL-1β (D) and IP-10 (E) concentrations were determined. (F) Cell viabilities were assessed using a MTT assay after supernatant collection. Results represent means ± S.E.M. from stimuli performed in duplicates and are representative of three different experiments. *, *P* ˂ 0.05; **, *P* ˂ 0.01, compared with LPS stimulus alone. (F) *, P<0.05 compared with the control without stimulus.

We analyzed whether compound **1** would also inhibit the production of other inflammatory mediators and the associated dose response. Macrophages were treated with different doses of compound **1** and stimulated with LPS. Treatment with compound **1** inhibited the production of NO, TNF-α, IL-6, IL-1β and IP-10 induced by LPS, in a concentration dependent manner (Figure 3, A, B, C, D, E), with an IC_50_ ranging from 2.75 ± 0.68 μM to 12.25 ± 0.40 μM (Table 1; Figure S2). The inhibitory effect of compound **1** was not due to its cytotoxicity, since concentrations between 12.5 μM and 25 μM did not significantly interfere with cell viability, as determined by the MTT method (Figure 3F and S3). 

**Table 1:** Compound 1 IC_50_ values for each inflammatory mediator.

	**IC_50_ ± S.D. (μM)**		
**Mediator**	**LPS**	**Pam_3_Cys**	**Poly I:C**
NO	7.54 ± 2.82	n.a.	n.a.
TNF	9.16 ± 0.83	5.28 ± 0.87	1.46 ± 0.11
IL-6	12.25 ± 0.40	8.04 ± 0.25	3.26 ± 0.94
IL-1β	2.75 ± 0.68	n.a.	n.a.
IP-10	3.73 ± 0.57	n.a.	7.10 ± 0.97

Values represent average of IC_50_ from three independent experiments ± S.D.

IC_50_ (positive control) = 1.47 ± 0.83 μM (value corresponds to the inhibition of NO production by the iNOS inhibitor L-N6).

n.a., not analyzed.

**Figure 4:**
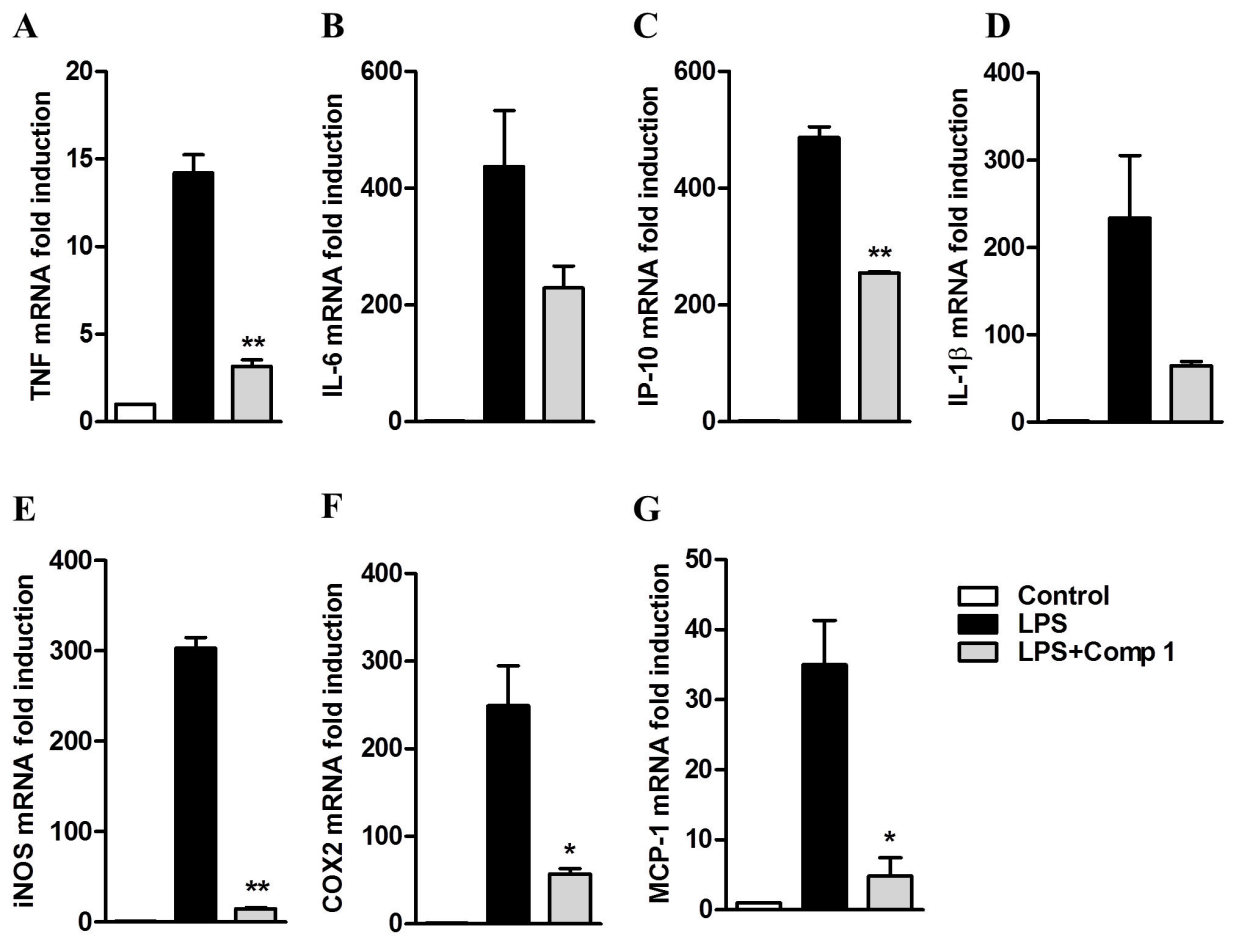
Expression of pro-inflammatory genes induced by LPS is inhibited by Compound 1. Peritoneal macrophages were treated with compound **1** (25 μM) and stimulated with LPS (1 μg/mL). After 180 min the RNA was isolated following the Trizol method. The amount of mRNA for TNF-α (A), IL-6 (B), IP-10 (C), IL-1β (D), iNOS (E), COX-2 (F) and MCP-1 (G) was determined by real-time RT-PCR. Results were normalized to HPRT expression and are presented as fold induction of mRNA expression relative to control samples. Results represent means ± S.E.M. from stimuli performed in duplicates and are representative of two different experiments. *, *P* ˂ 0.05; **, *P* ˂ 0.01, compared with LPS stimulus alone.

Analysis of mRNA levels in the cells treated with compound **1** and stimulated with LPS demonstrated a reduced induction of TNF-α (3x), IL-6 (2x), IP-10 (2x) and IL-1β (2x), indicating that compound **1** regulates the expression of these mediators at transcriptional levels (Figure 4, A, B, C, D). Despite the fact that the inhibition of IL-6 and IL-1β mRNA expression was not statistically significant, we clearly detected a conserved trend in several experiments, suggesting that the lack of statistical significance might be the result of low power due to our small sample size. We also measured the mRNA expression of iNOS, which is responsible for NO production in LPS-stimulated macrophages and found that iNOS mRNA levels were much lower in cells treated with compound **1** in comparison with the cells only stimulated with LPS (from 300 to 15 fold induction in non-treated and treated cells with compound **1** respectively). This suggests that the inhibitory effect on NO production may be associated with the regulation of iNOS gene transcription (Figure 4E). Finally, LPS-stimulated cells pretreated with compound **1** also showed decreased mRNA levels of COX2 (25x) and MCP-1 (4x) (Figure 4, F and G). 

As isogorgiacerodiol (Figure S1) was isolated from the same coral preparation as compound **1**, we also analyzed its effect on LPS-induced macrophage activation. Isogorgiacerodiol did not affect the production of TNF-α induced by LPS in macrophages, but inhibited NO production by these cells (IC_50_=15.86 ± 2.58 μM for isogorgiacerodiol versus 7.54 ± 2.82 μM for compound **1**; p =0.045). The IC_50_ values for IL-6 inhibition (12.25 ± 0.40 μM for compound 1 versus 21.56 ± 9.70 μM for isogorgiacerodiol) were not statistically different (p = 0.1105) (Figure S4). Taken together these results suggest that compound **1** has a higher inhibitory effect than isogorgiacerodiol.

### The Compound 1 Interferes with the Activation of NFκB but Not of MAPKs Induced by LPS

**Figure 5:**
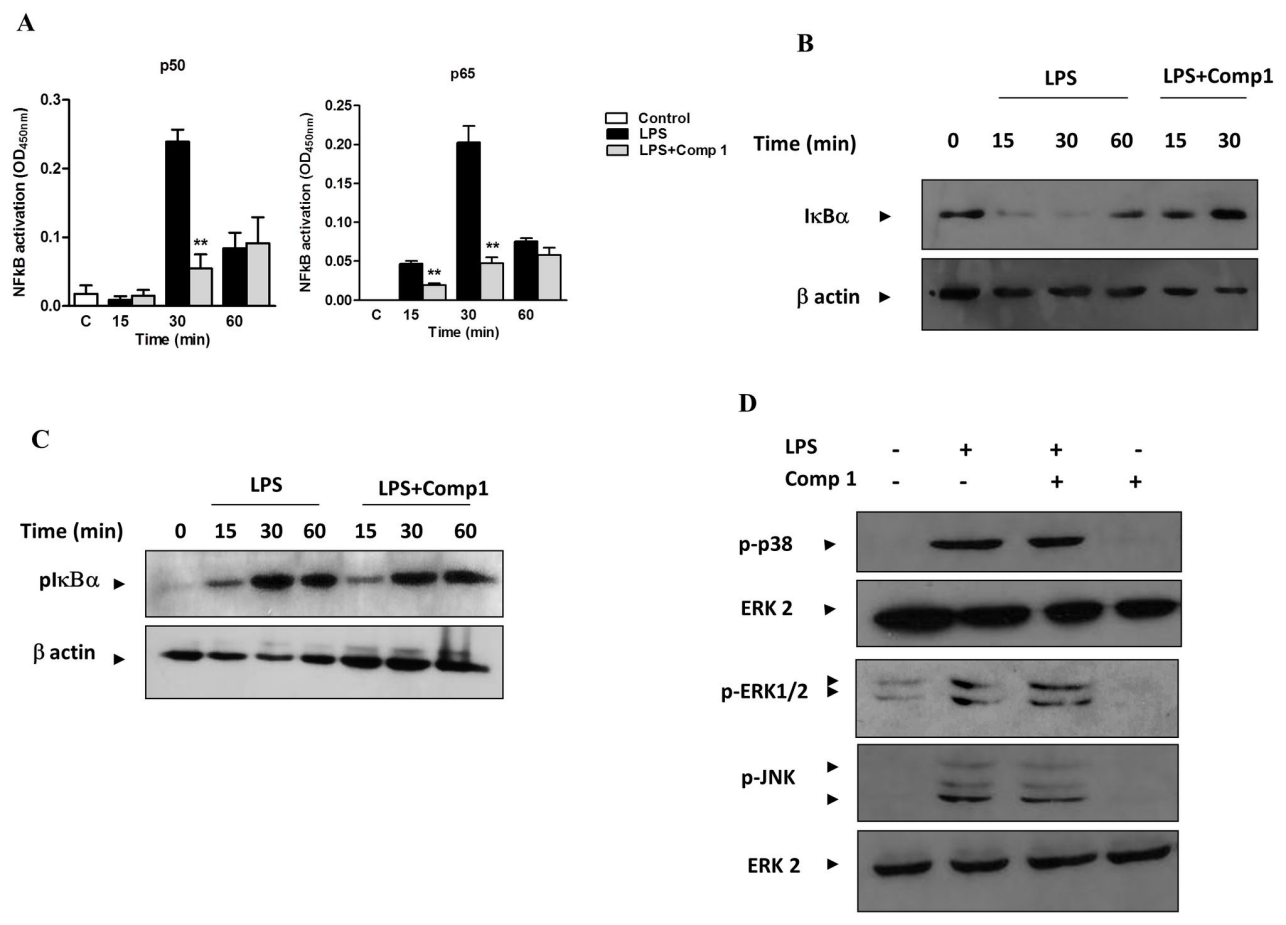
NF-κB activation induced by LPS is inhibited by compound 1. (A) Macrophages were treated or not with compound **1** (25 μM) and stimulated with LPS (100 ng/mL). After the indicated times nuclear extracts were prepared and the activation of p50 and p65 was evaluated using a TransAM assay. Results represent means ± S.E.M. from stimuli performed in duplicates and are representative of two different experiments. **, *P* ˂ 0.01, compared with LPS stimulus alone. Macrophages were treated as above and cell extracts were run in SDS-PAGE. IκBα degradation (B) and phosphorylation (C) was detected by immunoblotting. Detection of β-actin was used as loading control. The figures are representative of two different experiments with similar results. (D) Macrophages were stimulated for 30 min with LPS (100 ng/mL) in the presence or absence of compound **1** (25 μM). Cell extracts were submitted to SDS-PAGE. ERK1/2, JNK, and p38 phosphorylation was detected by immunoblotting. Detection of nonphosphorylated ERK2 was used as loading control. The figures are representative of two different experiments with similar results.

It has been previously shown that compounds from the diterpenes family have anti-inflammatory properties due to their capacity to inhibit the activation of NFκB at different levels [4]. Therefore, we investigated the effect of compound **1** in the NFκB activation pathway. Macrophages were treated with compound **1** and stimulated with LPS. The activation of NFκB (specifically the subunits p50 and p65) in nuclear extracts was evaluated by ELISA. LPS induced the activation of p50 and p65 after 30 min of stimulation, and this activation was significantly reduced by compound **1** (Figure 5A). 

Activation of NFκB requires its release from the IκBα inhibitor after phosphorylation of IκBα by the IKK complex, thereby inducing IκBα degradation. The degradation of IκBα was analyzed by western blot, and we observed that the treatment of macrophages with compound **1** prevented the IκBα degradation induced by LPS stimulation for 15 and 30 min (Figure 5B). On the other hand, we could not detect any difference in the levels of phosphorylated IκBα (Figure 5C), suggesting that the inhibitory effect of compound **1** is not associated with the activation of the IKK complex. 

Phosphorylation and activation of MAP kinases (MAPKs) is another event triggered by activation of macrophages by LPS, and it is essential for the production of inflammatory mediators [22]. Diterpenoids isolated from natural sources have been previously shown to inhibit the activation of MAPKs induced by LPS [23]. Therefore, we evaluated whether compound **1** would affect the phosphorylation of p-38, ERK 1/2, and JNK MAPK in LPS-stimulated macrophages. We did not observe any difference in MAPK phosphorylation in the cells treated or untreated with the compound, indicating that these pathways are not involved in the anti-inflammatory effect of compound **1**. 

### Compound 1 inhibits the activation of macrophages by other TLRs ligands and TNF-α

**Figure 6:**
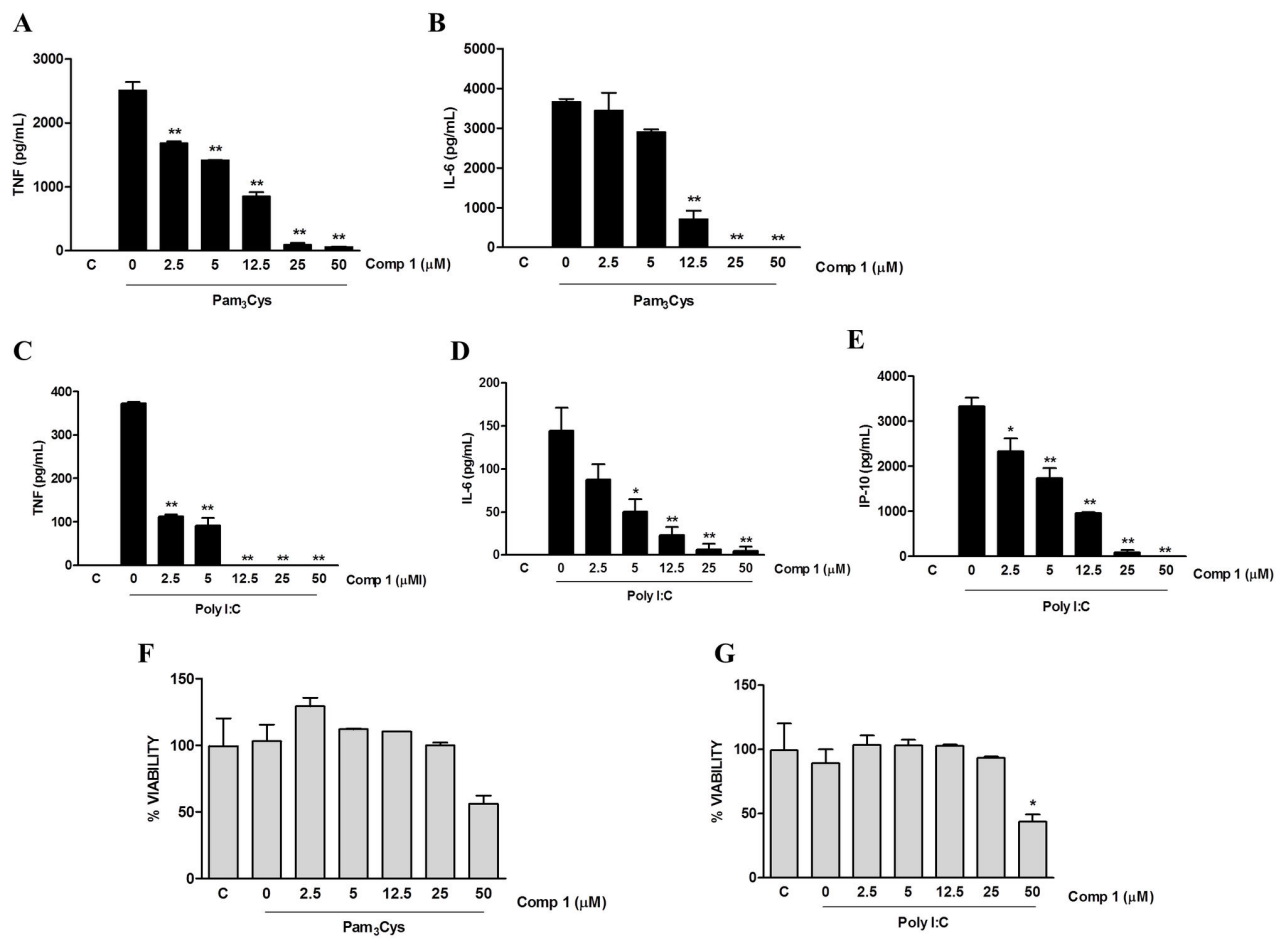
The production of pro-inflammatory mediators induced by other TLRs ligands is inhibited by Compound 1. Peritoneal macrophages were treated with the indicated concentrations of compound **1** (2.5, 5, 12.5, 25 or 50 μM). After 1 hour cells were stimulated with 100 ng/mL of Pam_3_Cys (A, B, F) or 20 μg/mL of Poly I:C (C-E, G). Supernatants were collected 24 h after the stimulus and TNF-α (A, C), IL-6 (B, D) and IP-10 (E) concentrations were determined. (F, G) Cell viabilities were assessed using a MTT assay after supernatant collection. Results represent means ± S.E.M. from stimuli performed in duplicates and are representative of three different experiments. *, *P* ˂ 0.05; **, *P* ˂ 0.01, compared with LPS stimulus alone.

Activation of NF-κB is a converging step in intracellular signaling pathways elicited by different stimuli, including the engagement of all TLRs and of cytokine receptors such as TNFR. Therefore, we examined whether the inhibitory effect of compound **1** would be expanded by other stimuli involved in macrophage activation. Peritoneal macrophages were stimulated by either TLR2 or TLR3 ligands (Pam_3_Cys and Poly I:C, respectively) in the presence or absence of compound **1** and the production of different inflammatory mediators was evaluated. Compound **1** inhibited, in a dose dependent manner, the production of TNF-α and IL-6 induced by Pam_3_Cys (Figure 6, A and B), with an IC_50_ of 5.28 ± 0.87 μM and 8.04 ± 0.25 μM, respectively (Table 1; Figure S5). The inhibitory effect of compound **1** was also observed on the secretion of TNF-α, IL-6 and IP-10 induced by Poly I:C (Figure 6, C, D, E), with IC_50_ values of 1.46 ±.0.11μM, 3.26 ± 0.94 μM and 7.10 ± 0.97 μM respectively (Table 1; Figure S5). These effects were not due to the cytotoxicity of the compound since at inhibitory concentrations cells were still viable (Figure 6, F and G). 

**Figure 7:**
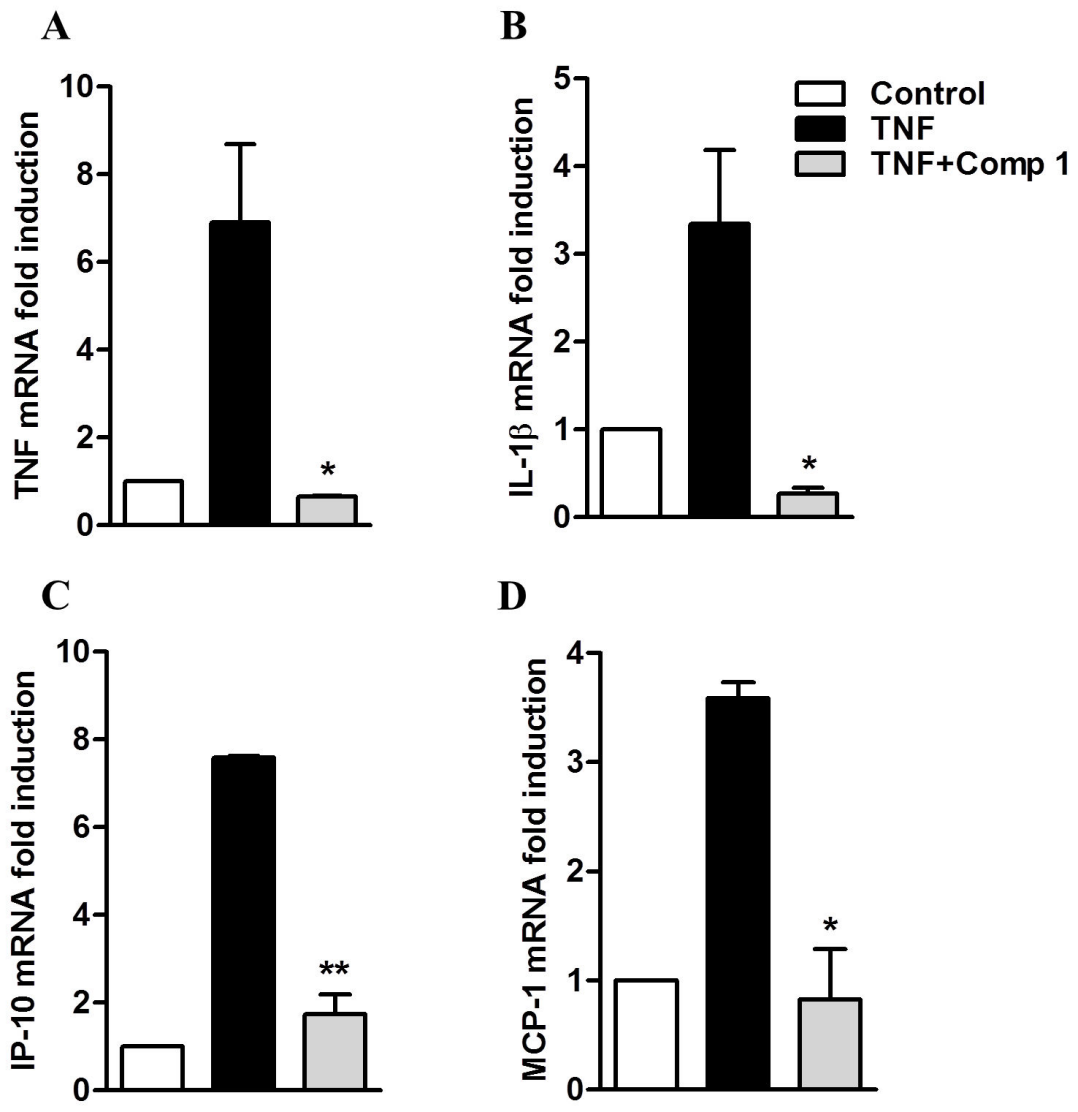
Compound 1 inhibits the expression of genes induced by TNF-α. Peritoneal macrophages were treated with compound **1** (25 μM) and stimulated with TNF-α (100 ng/mL). As a control, the cells were cultured with DMSO diluent. After 240 min of stimuli RNA was isolated following the Trizol method. The concentration of mRNA for TNF-α (A), IL-1β (B), IP-10 (C) and MCP-1 (D) was determined by real-time RT-PCR. Results were normalized to HPRT expression and are presented as fold induction of mRNA expression relative to control samples. Results represent means ± S.E.M. from stimuli performed in duplicates and are representative of two different experiments. *, *P* ˂ 0.05; **, *P* ˂ 0.01, compared with TNF-α stimulus alone.

TNF-α is produced by macrophages in many pathological conditions and has an autocrine and paracrine effect through its recognition by the TNF receptors (TNFR). TNFR engagement induces the recruitment of adaptor proteins, leading to activation of IKK and NFκB, which then stimulate the transcription of inflammatory genes. Since we observed that macrophage treatment with compound **1** decreased the mRNA levels of inflammatory mediators induced by LPS, we evaluated whether it would have the same effect on macrophage stimulation by TNF-α. Peritoneal macrophages were stimulated with TNF-α in the presence or absence of compound **1** and the levels of mRNA were determined by quantitative real time PCR. We observed that stimulation of macrophages with TNF-α induced the expression of TNF-α, IL-1β, IP-10 and MCP-1 mRNA (6, 4, 8 and 4 mRNA fold induction respectively), which were all abrogated when the cells were pretreated with compound **1** (Figure 7). 

### The expression of CD80 and CD86 induced by LPS is reduced by the treatment with compound 1

**Figure 8:**
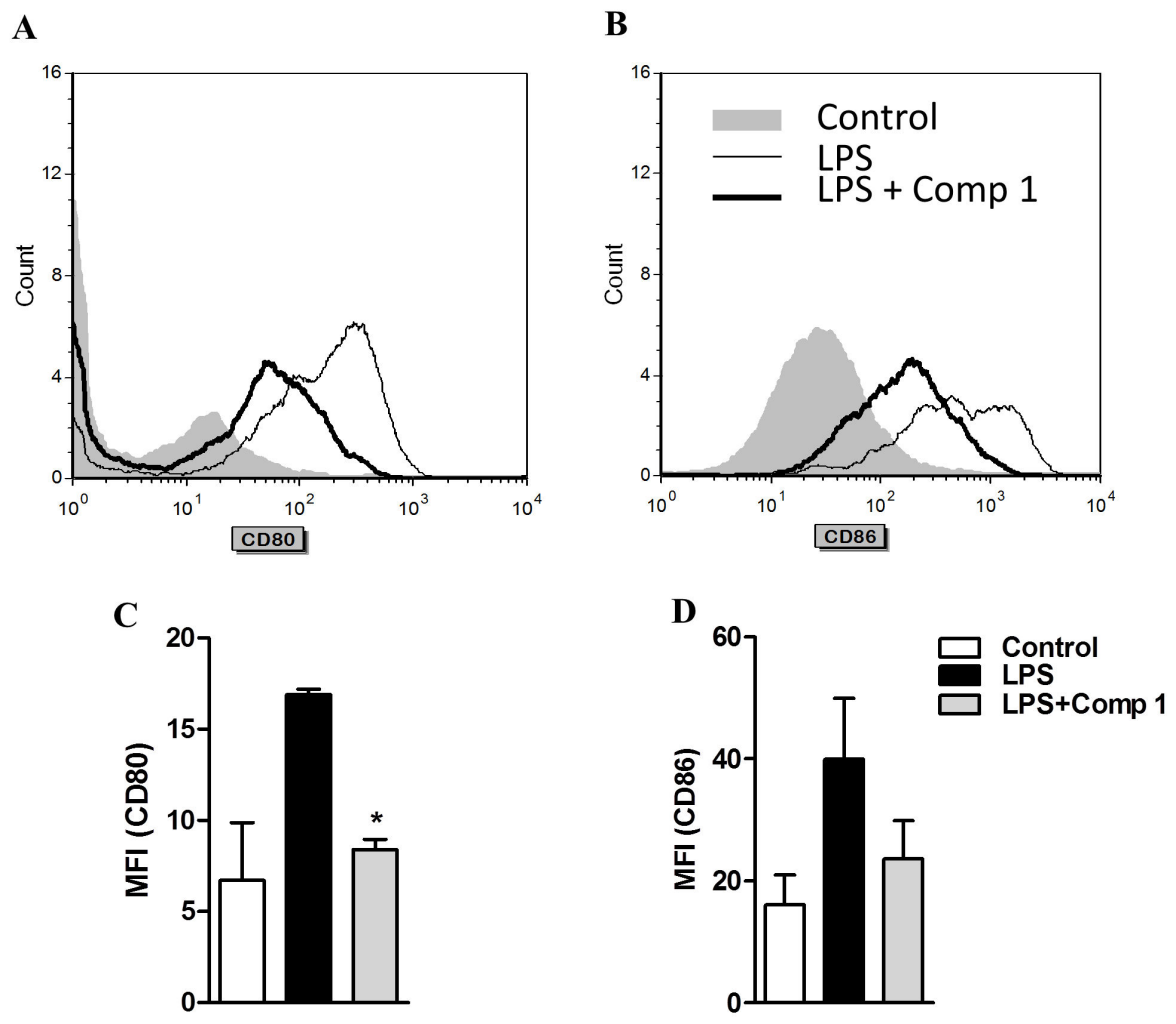
Compound 1 inhibits the expression of CD80 and CD86 induced by LPS in macrophages. Bone marrow derived macrophages were stimulated with LPS (1 μg/mL) in the presence or absence of compound **1** (25 μM). After 24 h cells were collected for FACS analysis of CD11b, CD80 and CD86 expression. Representative histograms of CD80 (A) and CD86 (B) on CD11b^+^ cells are shown from three different experiments. Graphics from CD80 (C) and CD86 (D) for the mean of fluorescence intensity on CD11b^+^ gated cells. Results represent means ± S.E.M. from two independent experiments. *, *P* ˂ 0.05, compared with LPS stimulus alone.

Activation of macrophages is also associated with several phenotypic changes, including the expression of the activation markers CD80 and CD86, which are essential for their co-stimulatory activity over adaptive immunity [24]. Thus, we assessed if compound **1** would have an effect on LPS-induced CD80 and CD86. Bone marrow derived macrophages were stimulated with LPS in the presence or absence of compound **1** and the expression of CD80 and CD86 was evaluated by flow cytometry. Macrophages treated with LPS showed a marked increase in the expression of CD80 and CD86 in comparison to non-stimulated cells (Figure 8). Interestingly, addition of compound **1** to the cultures strongly reduced the expression of both molecules, as measured by the decreased mean fluorescence intensities detected in the cells treated with LPS and compound **1** (Figure 8). These results indicate that compound **1** may modulate a variety of processes occurring during macrophage activation. 

## Discussion

In the present work we identified compound **1**, a diterpenoid isolated from the coral *P. acerosa*, as a potential anti-inflammatory molecule. Compound **1** inhibited the production of pro-inflammatory mediators induced by different TLRs ligands and TNF-α in macrophages. Our data suggest that this inhibitory effect was due to blocking of IκBα protein degradation and subsequent activation of the NFκB. 

Diterpenes are secondary metabolites that can be found in higher plants, fungi, insects and marine organisms and have the largest range of biological activities among the terpenoid family [4]. The anti-inflammatory characteristics of some members of diterpenoid family, especially those isolated from plants, have been described and mostly involve the inhibition of the NFκB signaling pathway at different levels [4]. Compound **1** was previously characterized as a methanol adduct of the diterpenoid pseudopterolide [20] and later isolated as a natural product [21], but its effect on inflammatory response had not been addressed previously.

We first evaluated the anti-inflammatory activity of compound **1** in macrophages’ response to LPS, which is a well characterized ligand of TLR4. The recognition of LPS by TLR4 leads to the recruitment of the adaptor proteins MyD88 (Myeloid differentiation primary response gene 88) and TRIF (TIR-domain-containing adapter-inducing interferon-β). The signal through MyD88 leads to the activation of NFκB and MAPKs, inducing the production of inflammatory cytokines, whereas the signal through TRIF induces the activation of IRF3, the induction of type I interferon and the late activation of NFκB [22]. All TLRs except TLR3 recruit MyD88, and TRIF is recruited by TLR3 and TLR4 [22]. The activation of NFκB through MyD88 and TRIF pathways depends on the degradation of IκBα after its phosphorylation by the IKK complex [7]. The gene transcripts induced after NFκB activation include pro-inflammatory mediators such as iNOS, COX-2, TNF-α, IL-1β, IL-6, MCP-1, and IP-10 among others [25].

We demonstrated that compound **1** inhibited, in a dose dependent manner, the production of inflammatory mediators such as NO, TNF-α, IL-6, IL-1β and IP-10 induced by LPS in macrophages. Moreover, compound **1** seems to have a higher inhibitory effect than isogorgiacerodiol, a known *P. acerosa* metabolite with very similar structure to compound **1** [21]. Compound **1** structurally differs from isogorgiacerodiol only by the presence of a methoxy group at C-9 (compound **1**) instead of the hydroxy group at same carbon (isogorgiacerodiol), suggesting that this functional group may be essential for the greater effectiveness of compound **1**. Previous reports support this possibility because it has been shown that subtle structural differences in small molecules are essential for modifying their immune modulation activities [26]. However, further studies are necessary to elucidate the molecular bases of the observed differences. 

Analysis of mRNA levels corresponding to inflammatory mediators demonstrated that compound **1** inhibited the LPS-induced transcription of TNF-α, IL-6, IL-1β, IP-10, MCP-1, and iNOS. Given that most of the non-steroidal anti-inflammatory drugs (NSAID) are inhibitors of COX2, we also analyzed COX2 mRNA levels. Interestingly, a significant reduction in the concentration of COX2 mRNA was observed, demonstrating that compound **1** has a broad effect on the transcription of pro-inflammatory genes associated with the LPS response. 

The inhibitory effect of compound **1** did not occur by competition with LPS for interaction with TLR4, since the inflammatory stimulus was inhibited even when compound **1** was added to the cultures after the addition of LPS. These results also suggest that compound **1** crosses the plasma membrane and achieves its effect by interacting with cytoplasmic signaling molecules. Diterpenoids from different families have hydrophobic characteristics that were previously associated with their ability to be incorporated in membranes [27-29]. Therefore, the nonpolar characteristics of compound **1** could also facilitate it crossing the plasmatic membrane. 

Several reports have demonstrated that diterpenoids from different natural sources act as inhibitors of the NFκB signaling pathway [30-32]. A Briarane, a diterpenoid isolated from the gorgonian octocoral *Briareum excavatum*, inhibited the cutaneous inflammation induced by TPA by interfering with the NFκB pathway [15]. Also, bharangin, a diterpenoid isolated from the medicinal plant *Premna herbacea*, inhibited the activation of NFκB by modifying residues on the p65 subunit and inhibiting the activation of IκBα [33]. Since NFκB regulates the expression of the pro-inflammatory mediators TNF-α, IL-6, IL-1β, IP-10, iNOS, COX2 and MCP-1 [25], we evaluated whether the anti-inflammatory effect of compound **1** would be attributed to inhibition of the NFκB signaling pathway. We demonstrated that compound **1** prevented the activation of the p50 and p65 subunits and the degradation of IκBα in LPS-stimulated macrophages. However, the phosphorylation of IκBα in response to LPS was not affected by compound **1**, suggesting that upstream components of the NFκB pathway are not affected. Compound **1** might be interfering with the ubiquitin - proteasome pathway, leading to the accumulation of p-IκBα. Inhibition of proteasome activity by terpenoids had been previously described and was associated with anti-inflammatory and/or anti-cancer functions [34,35]. Therefore, the role of compound **1** in the inhibition of ubiquitin - proteosome pathway needs to be investigated further. 

MAPKs are regulatory proteins involved in different signaling pathways triggered by activation of several innate immune receptors, such as TLR4. Four MAPKs cascades have been identified, including ERK1/2, JNK and p38 MAPK, and the activation of these cascades stimulated by TLR ligands in macrophages is involved in the induction of pro-inflammatory genes [22,36]. We did not observe any effect of compound **1** on the activation of ERK1/2, p38 and JNK induced by LPS in macrophages. Previous reports analyzing the effect of a marine diterpenoid of the briarane family in an *in vivo* model of TPA-induced dermatitis also demonstrated a selective inhibitory effect on NFκB activation with no interference with ERK1/2 activation [15]. 

Stimulation of an inflammatory response by macrophages can also be induced by other TLR ligands and by the engagement of cytokine receptors, such as TNFR. The cellular activation elicited by these receptors requires different adaptor molecules and triggers several initial signaling pathways, but most of them culminate in the activation of NFκB through the phosphorylation and degradation of IκBα [22,37,38]. We observed that compound **1** inhibited the production of pro-inflammatory mediators induced by TNF-α and by TLR2 (Pam_3_Cys) and TLR3 (Poly I:C) ligands, which are representative of the activation of MyD88-dependent and independent pathways, respectively. Further studies should be conducted to define whether this effect was also related to modulation of NFκB activation or was associated with upstream signals triggered by TNFR1 and TLR engagement. 

TLR2 is involved in the recognition of molecules derived from different pathogens such as bacteria, fungi, parasites and viruses. The production of inflammatory mediators induced by the activation of TLR2 depends on the recruitment of TIRAP and MyD88 adaptor molecules. TLR3 is essential in the recognition of virus dsRNA and specific small interfering RNA. Activation of TLR3 promotes the production of type I interferon and pro-inflammatory cytokines through the recruitment of the adaptor TRIF. Previous studies have shown the anti-inflammatory effect of various diterpenoids on the inhibition of the NFκB pathway triggered by TLR2 ligands [39]. The effect of the diterpenoid triptolide in the inhibition of the expression of genes induced by Poly I:C in macrophages has also been shown [40]. 

The activation of macrophages through PRR receptors induces the expression of major histocompatibility complex (MHC) and co-estimulatory molecules such as CD40, CD80 and CD86, which are critical for T cell activation [41]. Natural compounds have been described as modulators of the macrophage phenotype, which in turn affects the adaptive immune response [42,43]. We observed that compound **1** inhibited the expression of activation markers such as CD80 and CD86 induced by LPS in macrophages, suggesting that it may also interfere with the subsequent initiation of an adaptive immune response. 

In this work we demonstrated a previously unknown biological effect of the pseudopterane compound **1**. This molecule presented a relevant anti-inflammatory activity evidenced by a decrease in the expression and production of the pro-inflammatory mediators TNF-α, IL-6, IL-1β, IP-10, iNOS, COX2 and MCP-1 induced by LPS in macrophages without affecting cell viability. This activity was associated with its capacity to inhibit the degradation of IκBα and the subsequent activation of NFκB after LPS treatment. Compound **1** inhibited not only the response of macrophages to LPS, but also the response to TNF-α and ligands of TLR2 and TLR3. This compound also inhibited the expression of the co-stimulatory molecules CD80 and CD86 in response to LPS. Taken together, our results suggest that compound **1** might be a potential therapeutic agent for a variety of infectious and inflammatory diseases. 

## Supporting Information

Figure S1 **Schematic representation of isogorgiacerodiol.**
(TIF)

Figure S2 **Compound 1 inhibits the production of inflammatory mediators induced by LPS in murine macrophages.** IC_50_ sigmoidal curves calculated by the statistical software package GraphPad Prism 5 from the representative experiments shown in Figure 3. Graphs represent the sigmoidal curves for the IC_50_ calculation of NO (A), TNF-α (B), IL-6 (C), IL-1β (D) and IP-10 (E) induced by LPS in the presence of compound **1**. Results represent means ± S.D. from stimuli performed in duplicates.
(TIF)

Figure S3 **Compound 1 is not cytotoxic for macrophages.** Peritoneal macrophages from C57Bl/6 mice were treated with different concentrations of compound **1** (2.5, 5, 12.5, 25, 50 μM). After 24 h supernatants were collected and cell viabilities were assessed by a MTT assay. Results represent means ± S.E.M. from stimuli performed in duplicates.
(TIF)

Figure S4 **Isogorgiacerodiol inhibits the production of pro-inflammatory mediators induced by LPS in macrophages.** Peritoneal macrophages were treated with the indicated concentrations of isogorgiacerodiol (2.5, 5, 12.5, 25 or 50 μM). After 1 hour cells were stimulated with 10 ng/mL (A, B) or 1 μg/mL (C) of LPS. Supernatants were collected 24 hours after the stimulus and TNF-α (A), IL-6 (B) and NO (C) concentrations were determined. Results represent means ± S.E.M. from stimuli performed in duplicates and are representative of three different experiments. *, *P* ˂ 0.05; **, *P* ˂ 0.01, compared with LPS stimulus alone.
(TIF)

Figure S5 **Compound 1 inhibits the production of inflammatory mediators induced by Pam_3_Cys and Poly I:C in murine macrophages.** IC_50_ sigmoidal curves calculated by the statistical software package GraphPad Prism 5 from the representative experiments shown in Figure 6. (A, B) Sigmoidal curves for TNF-α and IL-6 induced by Pam_3_Cys in the presence of compound **1**. (C-E) Sigmoidal curves for TNF-α, IL-6 and IP-10 induced by Poly I:C in the presence of compound **1**. Results represent mean ± S.D. from stimuli performed in duplicates.
(TIF)
