# Supplementary figures and images for: A Pseudopterane Diterpene Isolated From the Octocoral Pseudopterogorgia acerosa Inhibits the Inflammatory Response Mediated by TLR-Ligands and TNF-Alpha in Macrophages
Source: PLoS One. 2013 Dec 16;8(12):e84107. doi: 10.1371/journal.pone.0084107 (PMC3865250; doi:10.1371/journal.pone.0084107)

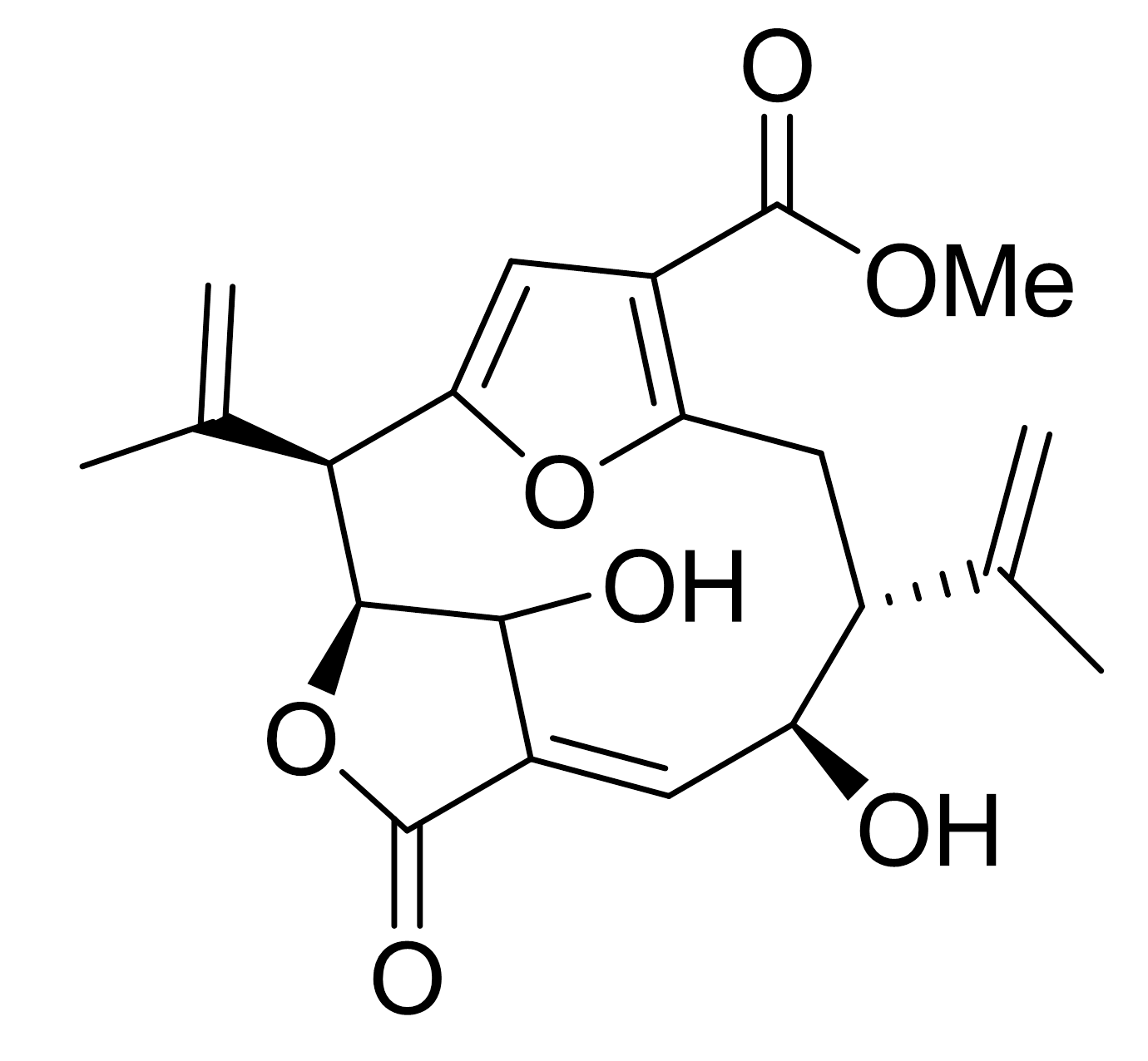

Supplement: Figure S1 — Schematic representation of isogorgiacerodiol. (TIF) [file pone.0084107.s001.tif]

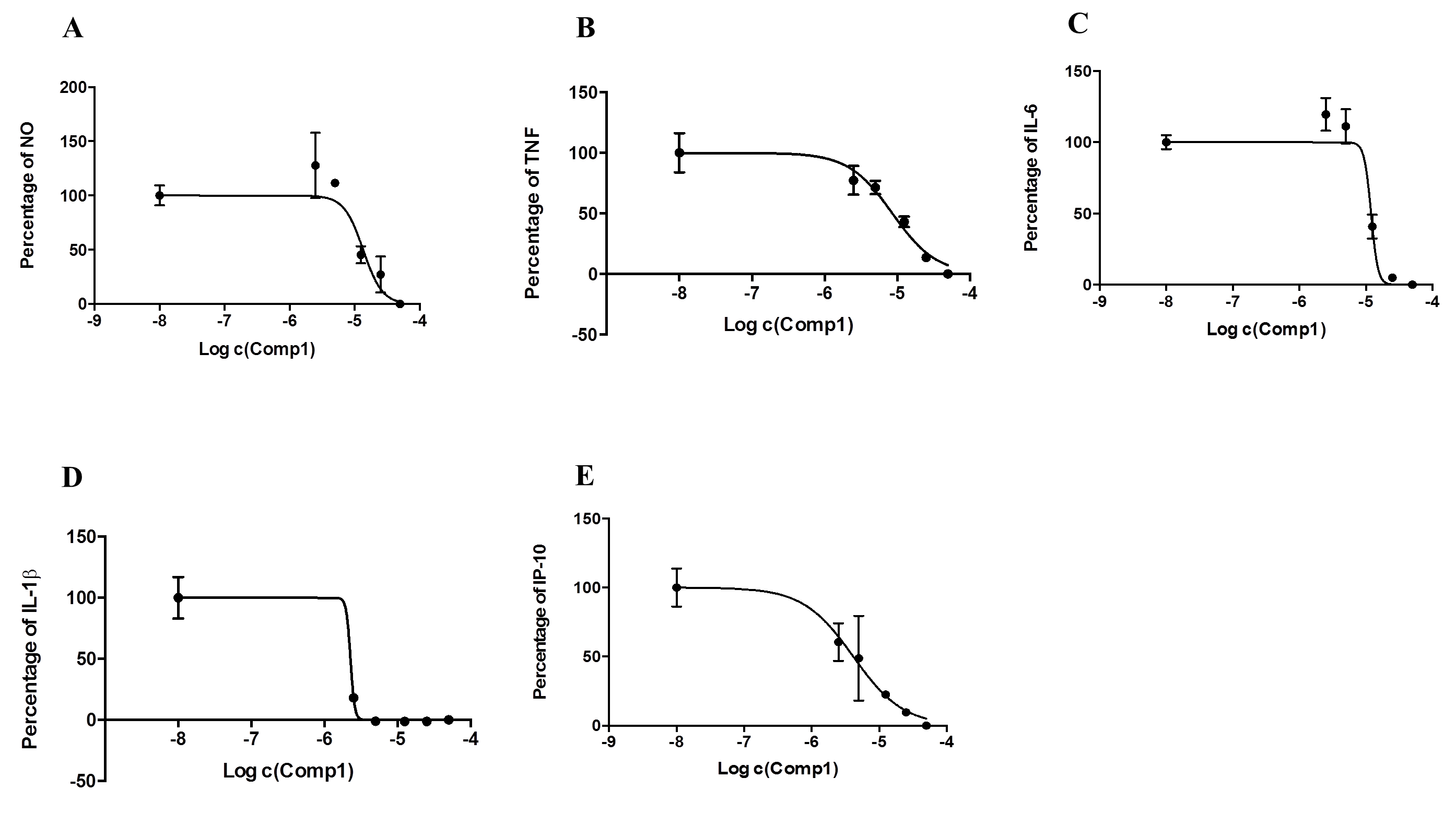

Supplement: Figure S2 — Compound 1 inhibits the production of inflammatory mediators induced by LPS in murine macrophages. IC50 sigmoidal curves calculated by the statistical software package GraphPad Prism 5 from the representative experiments shown in Figure 3. Graphs represent the sigmoidal curves for the IC50 calculation of NO (A), TNF-α (B), IL-6 (C), IL-1β (D) and IP-10 (E) induced by LPS in the presence of compound 1. Results represent means ± S.D. from stimuli performed in duplicates. (TIF) [file pone.0084107.s002.tif]

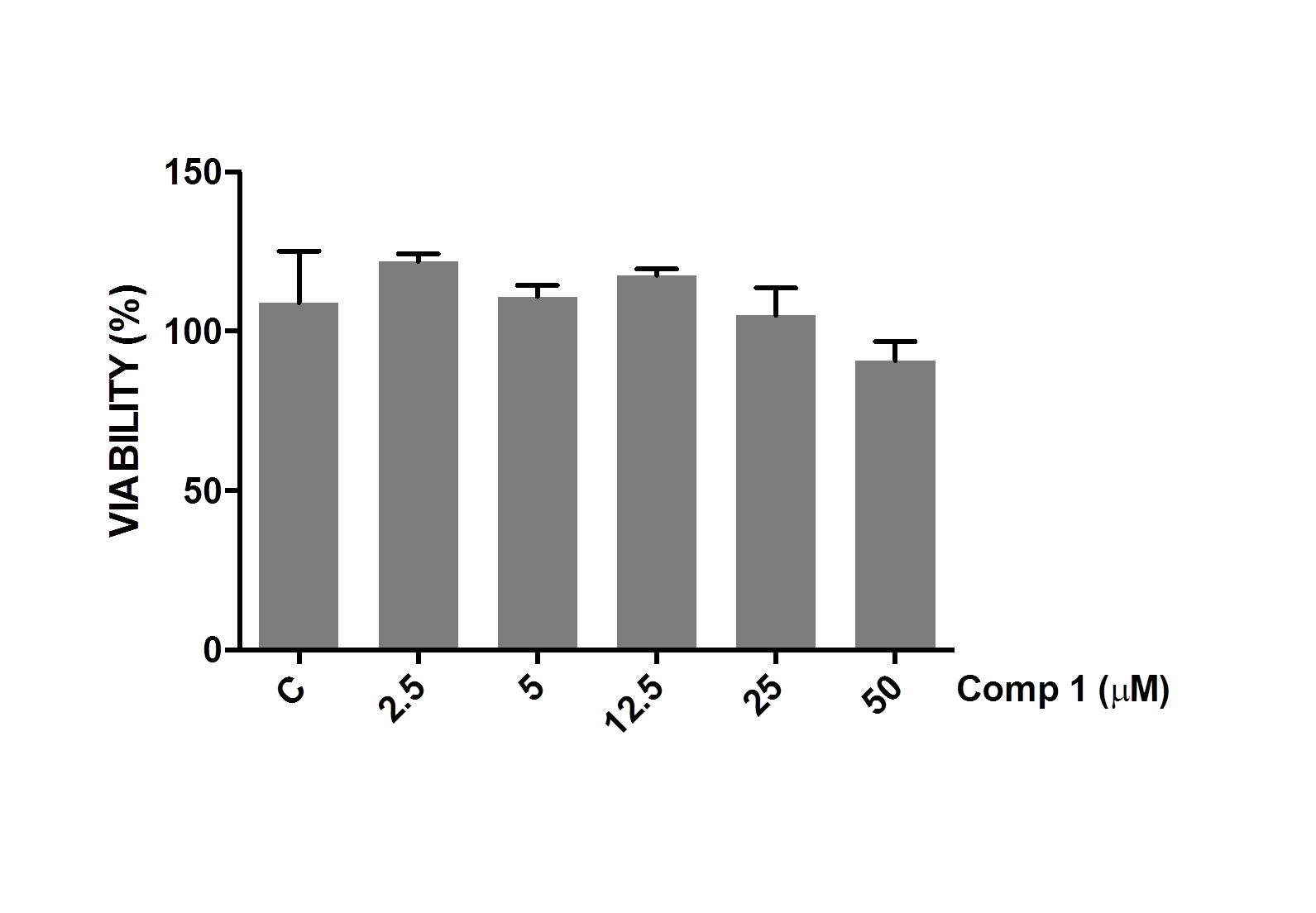

Supplement: Figure S3 — Compound 1 is not cytotoxic for macrophages. Peritoneal macrophages from C57Bl/6 mice were treated with different concentrations of compound 1 (2.5, 5, 12.5, 25, 50 μM). After 24 h supernatants were collected and cell viabilities were assessed by a MTT assay. Results represent means ± S.E.M. from stimuli performed in duplicates. (TIF) [file pone.0084107.s003.tif]

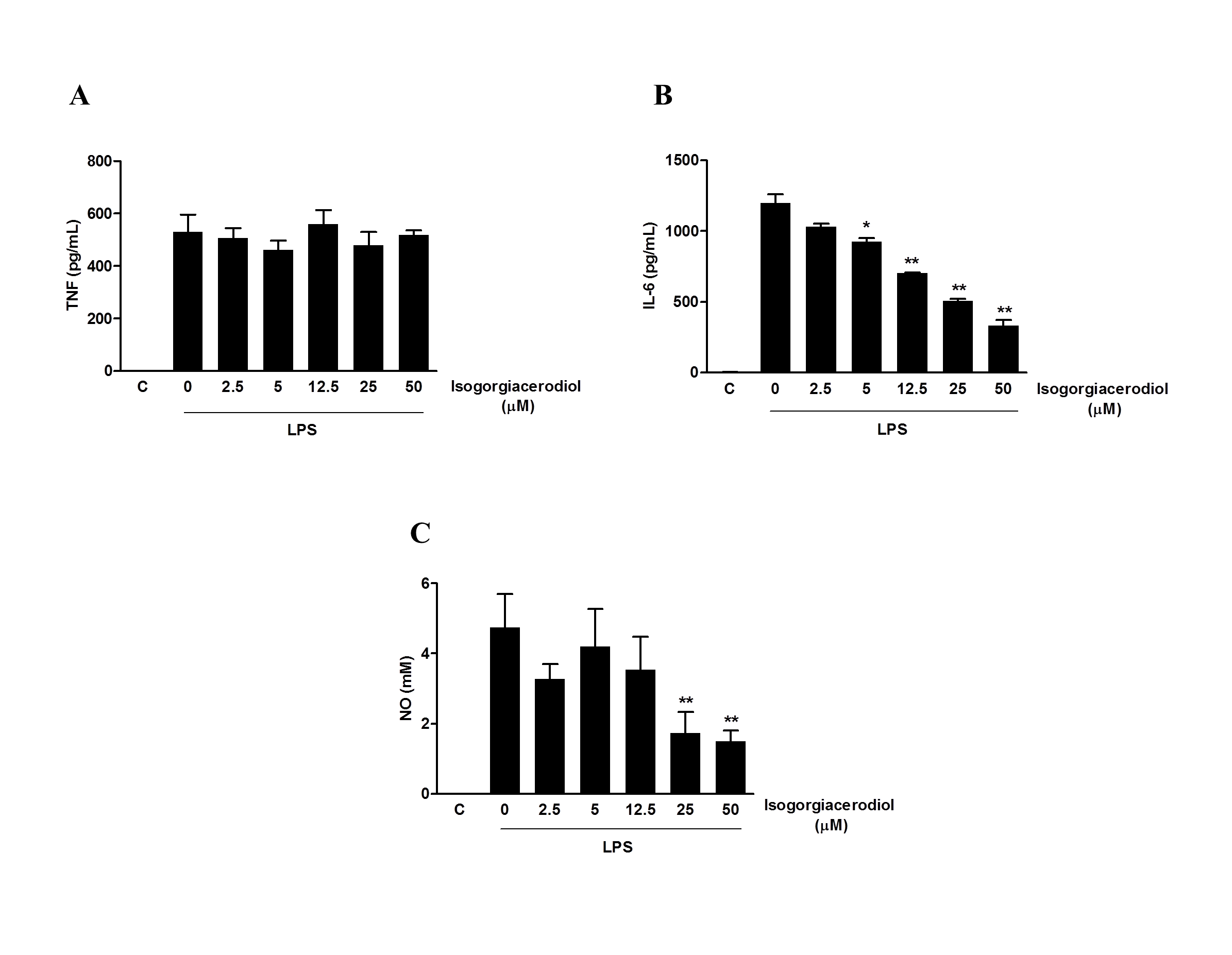

Supplement: Figure S4 — Isogorgiacerodiol inhibits the production of pro-inflammatory mediators induced by LPS in macrophages. Peritoneal macrophages were treated with the indicated concentrations of isogorgiacerodiol (2.5, 5, 12.5, 25 or 50 μM). After 1 hour cells were stimulated with 10 ng/mL (A, B) or 1 μg/mL (C) of LPS. Supernatants were collected 24 hours after the stimulus and TNF-α (A), IL-6 (B) and NO (C) concentrations were determined. Results represent means ± S.E.M. from stimuli performed in duplicates and are representative of three different experiments. *, P ˂ 0.05; **, P ˂ 0.01, compared with LPS stimulus alone. (TIF) [file pone.0084107.s004.tif]

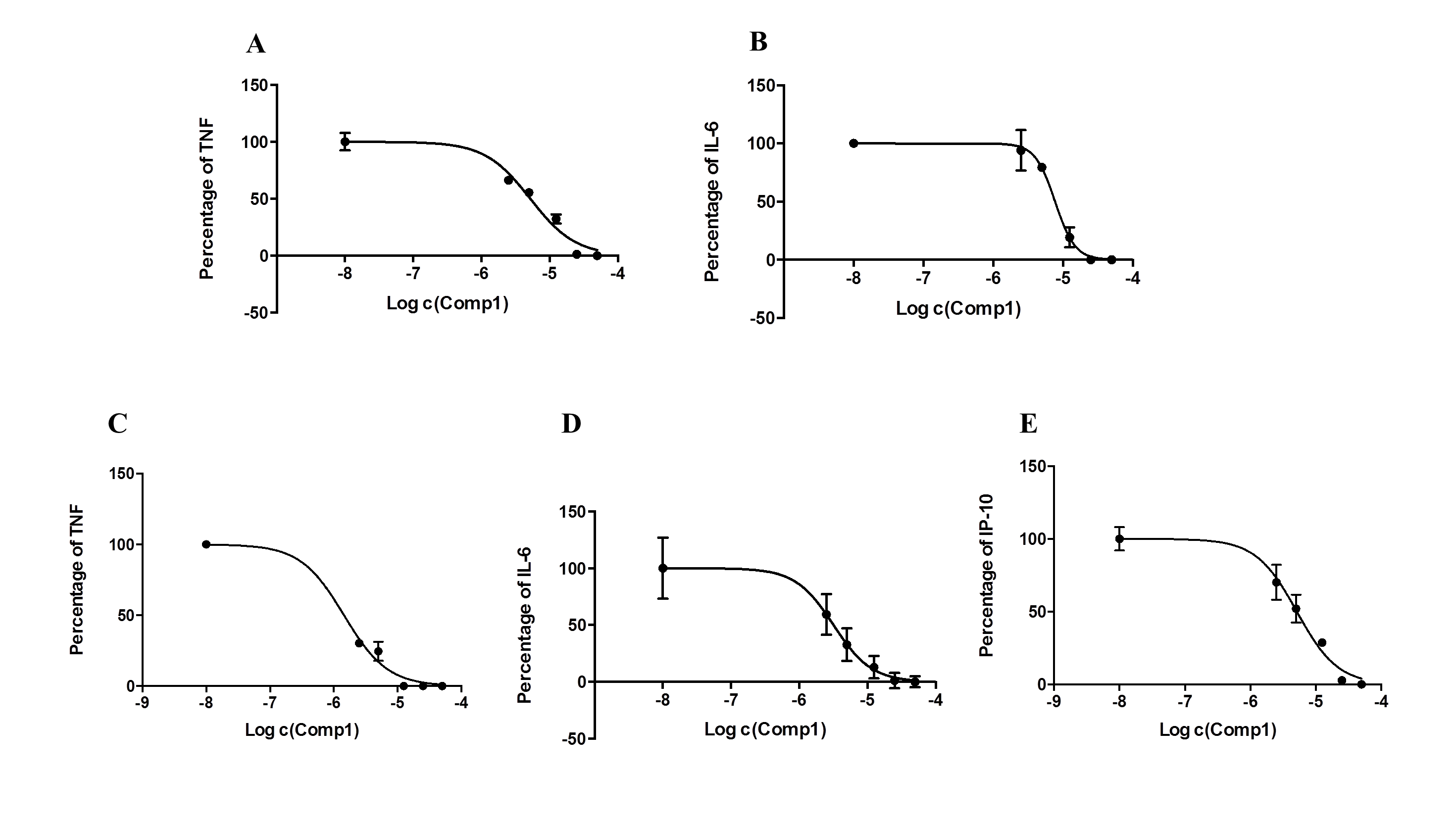

Supplement: Figure S5 — Compound 1 inhibits the production of inflammatory mediators induced by Pam3Cys and Poly I:C in murine macrophages. IC50 sigmoidal curves calculated by the statistical software package GraphPad Prism 5 from the representative experiments shown in Figure 6. (A, B) Sigmoidal curves for TNF-α and IL-6 induced by Pam3Cys in the presence of compound 1. (C-E) Sigmoidal curves for TNF-α, IL-6 and IP-10 induced by Poly I:C in the presence of compound 1. Results represent mean ± S.D. from stimuli performed in duplicates. (TIF) [file pone.0084107.s005.tif]
